# Temperature shapes the cold tolerance and body size of the springtail *Isotomiella minor* (Hexapoda, Collembola) in contrasting environments

**DOI:** 10.3897/zookeys.1277.172390

**Published:** 2026-04-09

**Authors:** Natália Raschmanová, Vladimír Šustr, Mária Fedičová, Michal Hurka, Martina Žurovcová, Ľubomír Kováč

**Affiliations:** 1 Department of Zoology, Institute of Biology and Ecology, Faculty of Science, Pavol Jozef Šafárik University, Šrobárova 2 SK-04154 Košice, Slovakia Institute of Soil Biology and Biogeochemistry, Biology Centre AS CR v. v. i. České Budějovice Czech Republic; 2 Institute of Soil Biology and Biogeochemistry, Biology Centre AS CR v. v. i., Na Sádkách 702/7, CZ-37005 České Budějovice, Czech Republic Institute of Entomology, Biology Centre AS CR v. v. i. České Budějovice Czech Republic; 3 Institute of Entomology, Biology Centre AS CR v. v. i., Branišovská 31, CZ-37005 České Budějovice, Czech Republic Pavol Jozef Šafárik University Košice Slovakia

**Keywords:** Body length, climate change, cold resistance, environmental factors, population ecology, soil fauna, springtails, traits

## Abstract

Urban environments exhibit narrower environmental temperature profiles, with increases in mean temperature compared with natural environments. In this context, urban populations will exhibit narrow thermal tolerance ranges, driven by a reduction in the ability to tolerate low temperatures and a more modest increase in the ability to tolerate high temperatures. Also, in the case of morphology, body size may vary with temperature in such urban-natural systems. Generally, there is a lack of knowledge on adaptive intraspecific variation in cold tolerance and body size of Collembola in these contrasting environments. In this study variation in these traits were explored in the common parthenogenetic springtail *Isotomiella minor* (Schäffer, 1896). The populations from warm urban habitats to tolerate lower temperatures less well than those inhabiting colder natural habitats was expected. Furthermore, whether the body size of populations may be related to habitat temperature was also explored. Significantly higher cold resistance was observed in the natural populations of *I. minor* compared to the populations from urban sites. The difference in the LD_50_ values between the populations also corresponded with their survival-temperature curves. The LD_50_ values correlated significantly with the soil temperature means at the sites. The body size of the *Isotomiella* populations decreased significantly with increasing soil temperature. Since elevation was the primary driver influencing temperature differences at sites, urbanisation effect could not be fully specified in our study. In conclusion, soil temperature, in combination with other environmental factors, plays a crucial role in the cold tolerance and body size of *Isotomiella minor* in urban and natural environments.

## Introduction

Intraspecific thermal tolerance and body size variations are essential to comprehensively understanding of ecological and evolutionary responses of populations of the species to environment. Such characteristics or traits (defined as characteristics that mediate responses to the environmental conditions) may help to shed more light on the ecological and evolutionary responses of populations to climate changes; cities in particular are on the frontline of climate change. Recent studies from anthropogenically contrasting environments by Blake et al. (1994), Magura et al. (2004), Sadler et al. (2006), Angilletta et al. (2007), Diamond et al. (2017) and Yilmaz et al. (2021) have documented in selected soil invertebrate taxa such as ants, beetles, and isopods that these crucial eco-physiological and morphological features are strongly linked to environmental conditions and to urbanisation, which is usually associated with changing temperatures. In Collembola, an abundant and diverse component of soil fauna (Hopkin 1997), such studies focused on adaptive intraspecific traits in thermal tolerance and body size from anthropogenically contrasting environments are scarce (Escribano-Álvarez et al. 2022), and the lack of them is surprising given that several springtails are commonly found in urban areas (Fountain and Hopkin 2004; Potapov et al. 2023). Specific characteristics of urban and natural environments provide unique living laboratories for investigating patterns of intraspecific variation and also testing general hypotheses in thermal ecology and body size of invertebrates (Diamond et al. 2015). In Collembola, only intraspecific trait variations in thermal tolerance across environmental (latitudinal) gradients have been documented (Bahrndorff et al. 2006, 2009). These studies showed that populations of the same species from environments with variable temperature have a broader thermal tolerance span compared to populations inhabiting thermally stable environments, which is in agreement with the climatic variability hypothesis (Stevens 1989). Raschmanová et al. (2018) found a similar pattern when testing this hypothesis in Collembola along a surface-subterranean habitat and temperature gradient. Regarding the relevance of this hypothesis in the contrasting environments, such as urban and natural sites, anthropogenic factors often result in elevated temperatures in the regional climate of urban environments compared to natural sites (e.g., Roth 2002). Previous studies (e.g., Imhoff et al. 2010; Stewart and Oke 2012; Diamond et al. 2015) have suggested that temperature profiles in urban-natural gradients are parallel with those in latitudinal gradients, i.e., that urban areas (similarly as in low latitude areas) exhibit narrower environmental temperature ranges with increases in mean temperature compared with rural areas (similar to high latitude areas). We may then expect the microgeographic pattern in urban-natural systems to be similar to the macrogeographic pattern in latitudinal gradients, i.e., urban populations will exhibit narrow thermal tolerance ranges, driven by a reduction in the ability to tolerate low temperatures and a more modest increase in the ability to tolerate high temperatures. However, empirical studies supporting this theory, conducted along urbanisation gradients, are still scarce (Angilletta et al. 2007; Diamond et al. 2015). Thus, we conclude that climatic variability is the ultimate driver of both intra- and interspecific variation in thermal tolerance across such environmental gradients with climatically contrasting habitats. It has been observed that elevational gradients also significantly affect the body size of Collembola populations, which is associated with the changing environmental temperature (Sun et al. 2020a, 2020b; Xie et al. 2022, 2024). One of these studies showed that body size increased with increasing elevation (and also with decreasing temperature) in tropical montane rainforests. This relationship in ectothermic invertebrates is referred to as Atkinson’s temperature-size rule (Atkinson 1994), or as Bergmann’s rule sensu lato (Bergmann 1847). Habitat temperature affects body size through developmental and metabolic rates (Atkinson 1994). Ectotherms are likely to get larger under lower temperatures since their metabolic rate decreases and consuming additional resources is needed to compensate for this decrease (Atkinson 1994). However, several studies including different taxa, have come to inconsistent conclusions in this context (Shelomi 2012), and also in Collembola (Sun et al. 2020a). This relationship is species-specific and depends partially on the study design.

The springtail *Isotomiella minor* (fam. Isotomidae) is a small edaphic species characteristic with parthenogenic reproduction and a broad Holarctic geographic distribution beyond the high Arctic (Potapov 2001). As a eurytopic species, it inhabits a variety of open and forest habitats, which is testimony to its wide ecological plasticity. It prefers humid forest soils and is less abundant in grasslands, meadows, and urban areas (Kuznetsova 2006; Fjellberg 2007; Fiera 2009; Santorufo et al. 2014; Heiniger et al. 2015; Raschmanová et al. 2016). This isotomid is classified as a mesohygrophilous species (Kuznetsova and Krestyaninova 1998). Despite *Isotomiella minor* being well characterised morphologically and ecologically, its thermal tolerance (Agrell 1941; Zettel 1984) and body size remain less known. In this context, an examination of ecophysiological and morphological traits in *I. minor* is highly required. The specific environmental conditions in urban-natural systems may shape these traits (Diamond and Martin 2016; Diamond et al. 2017), thus appearing as suitable habitat complexes for such type of studies.

The aim of the present study was to examine how temperature may affect the cold tolerance and body size of *Isotomiella minor* populations inhabiting contrasting habitats of urban and natu­ral environments in Central Europe. Two contrasting ecosystems were selected: a city agglomeration as an urban environment and karst landscape as a natural environment. These environments differed in basic climatical and topographical conditions. We hypothesised that (1) populations of *Isotomiella minor* from the contrasting habitats could show different cold tolerance as a result of physiological adaptations of the local populations to specific site characteristics. Furthermore, we applied the climatic variability hypothesis to the thermal tolerance of *Isotomiella* populations that inhabit contrasting urban and natural environments. We expected that (2) urban populations occupying environments with less fluctuating temperatures and higher means will tolerate lower temperatures less well than those inhabiting colder natural habitats. We further expected that (3) populations of *I. minor* in such contrasting environments could show different body length; therefore, we sought to determine whether the body size of populations is somehow related to habitat temperature.

## Materials and methods

### Collection dataset

**Figure 1.**
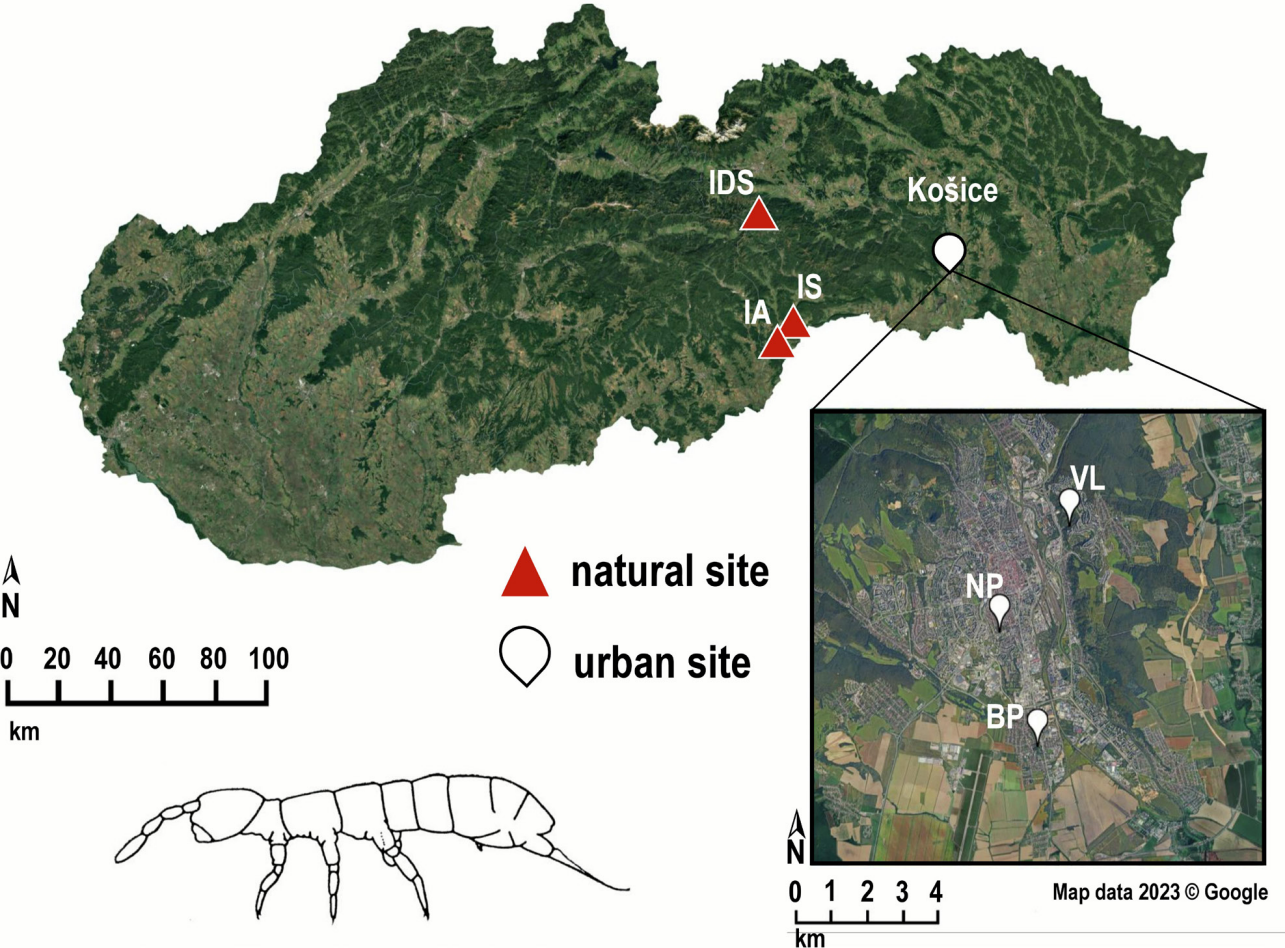
Location of the natural and urban sites with the studied *Isotomiella minor* populations in Slovakia. For abbreviations of sampling sites, see the Materials and methods section. *Isotomiella minor*, drawing according to Gisin (1960).

Six populations of *Isotomiella minor* were selected from two contrasting ecosystems composed of natural and urban habitats. Populations inhabiting habitats with a distinct level of anthropogenic effects served for this study, namely two city parks (BP, NP) and a fragment of a woodland (VL) in an urban agglomeration of the city of Košice (~ 240 km^2^ and ~ 239,000 inhabitants). The natural populations came from sites in a karst landscape located near cave entrances in the Slovak Karst (IA, IS) and Slovak Paradise (IDS) national parks in the Western Carpathians, Slovakia (Fig. 1). Internal cave microclimate did not affect substantially the associated forest sites, which were situated in front of entrances to caves. The maximum geographic distance between the urban and natural site was ~73 km (BP– IDS). The term “populations” of *Isotomiella minor* is used in this study, but these populations actually represented different cryptic lineages, or even one such “population” represented a mixture of different cryptic lineages (Fedičová et al. 2025). Nevertheless, we state that study traits with observed genetic differences cannot be directly related in the present study, since different specimens of the same population were tested for molecular and physio-/morphological parameters.

For the topographic, vegetation and soil-chemical characteristics of the sites with the studied *Isotomiella* populations, see Table 1. Data on soil organic carbon content at the studied sites were added from previous studies (Jureková et al. 2019; Marcin et al. 2021) and unpublished sources. Organic carbon content (from four depth layers per site) was analysed using the dynamic flash combustion method (Carter and Gregorich 2008) at the Soil Science and Conservation Research Institute in Bratislava.

**Table 1.** Characteristics of urban and natural sites.

Sampling site	Coordinates	Elevation m (a.s.l.)	Vegetation and soil organic carbon content (C_org_, %)
**Urban**			
BP – Park (Košice-Barca)	48°40'42.466"N, 21°16'1.115"E	196	park trees with *Tilia cordata* and *Acer plantanoides*, sparse herbal cover, litter cover: 0-1 cm, C_org_: 3.23
NP – Park (Košice)	48°42'25.737"N, 21°15'11.821"E	203	park trees with *Gleditsia triacanthos* and *Acer plantanoides*, sparse herbal cover, litter cover: 0 cm, C_org_: 2.49
VL – Fragment of woodland (Košice)	48°43'59.546"N, 21°16'49.022"E	247	thermophilous oak wood, dense bush, sparse herbal cover, litter cover: 1 cm, C_org_: 4.91
**Natural**			
IA – In front of the cave entrance, Ardovská Cave (distance to cave entrance ~ 45 m)	48°31'16"N, 20°25'14"E	317	thermophilous cornel-oak wood (ass. *Corneto*–*Quercetum*), scree slope (SW exposition, 20° slope), mosses and lacking herbal cover, litter cover: 2 cm, C_org_: 12.20
IS – In front of the cave entrance, Silická ľadnica Ice Cave (distance to cave entrance ~ 77 m)	48°32'55"N, 20°30'12"E	480	thermophilous hornbeam wood (ass. *Waldsteinio*–*Carpinetum*), scree slope (W exposition, 35° slope), middle-dense herbal cover, litter cover: 2 cm, C_org_: 11.50
IDS – In front of the cave entrance, Dobšinská Ice Cave (distance to cave entrance ~ 17.3 m)	48°52'05"N, 20°18'14"E	969	coniferous wood (ass. *Fageto*–*Piceetum*), scree slope (N exposition, 30° slope), dense herbal cover, litter cover: 3-4 cm, C_org_: 23.72

Regarding climatic data, the city of Košice is characterised by a mean annual air temperature of +8.7 °C with annual average precipitation of 605 mm (Kaya et al. 2021). The Slovak Karst region is characterised by a mean annual air temperature ranging from +5.7 to +8.5 °C and annual average precipitation from 630 to 990 mm (Rozložník et al. 1994). In Slovak Paradise National Park the mean annual air temperature ranges from +4.7 to +6.4 °C with annual average precipitation from 648 to 954 mm (Huňa et al. 1985).

Continual measuring of soil temperature was done from May 2022 to May 2023. The basic temperature characteristics of the selected urban and natural sites (BP, NP, VL and IA, IS, IDS) were measured, i.e., maximum, minimum and mean soil temperatures (T_max_, T_min_, and T_mean_), including both the 6-month vegetation period and annual temperature data (with exception of site NP, where only a 6-month period was available). The soil temperature was measured continually every 4 h (iButton DS1921G data-loggers, with an accuracy of ± 0.5 °C) at each site at a soil depth of 3 cm.

Logistically, we were unable to sample animals from urban and natural sites at the same day. However, soil sampling was completed within the same week (urban BP, NP, VL: 4 November 2022 and natural IA: 8 November, IS, IDS: 10 November 2022). The soil samples were collected at selected sites using a soil corer 10 cm in diameter to a maximum depth of 8–10 cm. Collembola specimens were extracted alive from soil samples using a modified high-gradient apparatus (Crossley and Blair 1991). For the morphological study, extracted specimens were fixed in pure ethyl alcohol. The total number of adult specimens per each population was the following: BP – 76 specimens, NP – 65 specimens, VL – 74 specimens, IA – 85 specimens, IS – 80 specimens and IDS – 83 specimens. The larger portion of the extracted material was used for the cold tolerance experiment to determine lethal temperatures (see below). Regarding soil sampling, the research adhered to the conditions of License #2661/2017-6.3. from the Ministry of the Environment of the Slovak Republic.

### Cold tolerance testing and body size measurements

All pre-exposure manipulations (before testing) and the experimental protocol followed the studies of Raschmanová et al. (2015, 2017, 2018), using the methodology for cold tolerance testing of natural (non-laboratory) populations of Collembola. After field sampling, the soil samples were stored in a cool bag and transported the same day to the laboratory. During extraction the specimens were collected alive and subsequently transferred to laboratory cultures and stored at +10 °C (approximate temperature of their habitats). Specimens were stocked in small plastic boxes containing a moist plaster substratum as a source of water. Plaster of Paris was watered when necessary to keep it moist. Specimens were not fed before or during the experiment. The animals were kept ~10–12 h at these conditions before the experiment. All these pre-exposure manipulations and cold tolerance testing were identical across all populations. Cold tolerance experiment began approximately ~24 h after field sampling. A total of 317 specimens (49–57 specimens of each population) were tested for cold tolerance (Table 2). The 50% lethal dose (LD_50_), defined as a temperature at which 50% of the exposed individuals of a species do not survive at 1 h of exposure, was used as a parameter for thermal tolerance quantification. A short exposure time (1 h) was chosen to investigate the differences in survival of populations since we were not focused on testing a whole range of exposure times on the survival of individuals. Within such a design, these measurements were carried out in a reasonable time, as it was necessary to ensure that the changes in temperature before exposure and the return to temperature occurred at a constant rate were comparable to other cold hardiness experiments. The survival of individuals was tested in 1.5-ml Eppendorf tubes, with one individual placed on the surface of the plaster of Paris (0.1 ml of water-saturated plaster to maintain high moisture) covering the bottom of the tube. Testing one individual at a time instead of groups of individuals was advantageous, because testing multiple individuals in one tube could mask individual variability, as the interaction of multiple individuals could affect the result in different ways (e.g., mechanical disturbing, sharing of nucleating agents affecting supercooling). The tubes were closed and inserted into the block of the laboratory cooling–heating thermostat (CH-100, BIOSAN, Latvia). The correct temperature on the surface of the plaster in the Eppendorf tubes and the time needed for temperature equilibration were verified using a miniature bead thermistor (NR 506 70 K, PMEC Šumperk) in a series of preliminary measurements.

**Table 2.** Temperature characteristics of urban and natural sites and number of *Isotomiella minor* specimens used in the temperature tolerance experiment and body size (length) measurements.

Sampling site	(a) T_mean_ ± SD	(m) T_mean_ ± SD	Cold tolerance N	Body length N
	T_min_ – T_max_ (°C)	T_min_ – T_max_ (°C)		
**Urban**				
BP – Park (Košice-Barca)	10.7 ± 6.9	16.5 ± 3.9	51	25
	0–25	8.5–25		
NP – Park (Košice)	–	16.3 ± 3.2	49	16
		10–22.5		
VL – Fragment of woodland (Košice)	10.2 ± 5.5	15.1 ± 2.7	50	24
	0–20.5	10–20.5		
**Natural**				
IA – In front of the cave entrance, Ardovská Cave	9.6 ± 5.6	14.4 ± 3.0	55	30
	0–20.5	9–20.5		
IS – In front of the cave entrance, Silická ľadnica Ice Cave	8.5 ± 4.8	12.1 ± 2.3	55	30
	0.5–17.3	8.3–17.3		
IDS – In front of the cave entrance, Dobšinská Ice Cave	4.6 ± 2.8	7.1 ± 1.1	57	26
	0.5–9.5	3–9.5		

a – annual mean soil temperature (11 May 2022–10 May 2023), m – 6-month mean soil temperature (11 May 2022–10 November 2022): T_mean_ – mean temperature, T_min_ – daily minimum temperature, T_max_ – daily maximum temperature, SD – standard deviation, N – number of specimens used in the cold tolerance experiment and body length measurements.

Cold survival was tested with a starting temperature of +10 °C; the tubes containing Collembola were then gradually cooled in 4-min steps. The tested temperature was obtained by cooling at a constant rate (0.15 °C per minute). The tube was exposed to the tested temperature for one hour. The temperature was then brought back to the initial temperature at the same rate, and the survival of each individual was tested. An individual who survived the experiment was an individual in whom some movements were observed after the experiment and also after a 24-h recovery period at high air humidity and a temperature of 12 °C. The range of tested temperatures was established after preliminary range-finding tests. Cold survival was tested in a temperature range from –2.4 to –7.2 °C in 0.6 °C steps (7–9 temperatures), in agreement with Sinclair et al. (2015) suggesting a need of ~ 5 temperature values spanning the range of 0–100% mortality (in terms of successful determination of lethal temperatures and strength/significance of results).

Due to sampling difficulties in the field and in experimental testing, obtaining such physiological data was not trivial (Raschmanová et al. 2017; Escribano-Álvarez et al. 2022). During testing, careful handling of the specimens was required due to their minute body size (≤1 mm) and sensitivity to low air humidity. Therefore, the number of specimens used at the tested temperatures in the cold tolerance analysis was dependent on their availability. Only vital specimens without visible damage were selected for the experiment. However, their number was still comparable with other thermal tolerance studies with the field populations (e.g., Mammola et al. 2019; Colado et al. 2022; Isaia et al. 2022). A total of 5–13 specimens of *Isotomiella minor* were tested at each temperature (BP: 6–13 specimens, NP: 6–9 specimens, VL: 6–9 specimens, IA: 5–7 specimens, IS: 5–7 specimens, IDS: 5–8 specimens). After the experiment, each specimen was fixed in ethyl alcohol for species verification using a Carl Zeiss Axiolab A1 phase-contrast microscope (Carl Zeiss Microscopy, Oberkochen, Germany). All temperature experiments were carried out at the Institute of Biology and Ecology, Faculty of Science, P. J. Šafárik University (IBE FS PJSU), Košice, Slovakia.

For the morphological study, the extracted specimens were separately mounted on permanent slides in Swann medium (Liquido de Swann) modified after Rusek (1975) and measured using a Carl Zeiss Axiolab A1 phase-contrast microscope. The body size measurements were performed with a micrometric eyepiece. Body length was measured on slides from the head to the end of abdomen, excluding appendages. All specimens were carefully assessed for damage that would prevent accurate measurements. For measurement precision, two collembolan specialists performed the measurements independently. Measurements were taken in random order and blind to information on the site of origin. Adult individuals were identified by the presence or absence of fully developed genital area. A total of 151 adult specimens (16–30 specimens of each population) were used for the body size measurements (Table 2). The collection of *Isotomiella minor* specimens from the study sites is available at the IBE FS PJSU.

### Cold tolerance and body size data analyses

Statistical analyses followed Raschmanová et al. (2018). In short, the GLM binomial model (R v. 3.3.1.) was used to test the impact of temperature on the relative frequency of survival and the temperature response of the species (Venables and Smith 2009). The model used the logistic curve y = exp(a + b*T) / (1 + exp(a + b*T)), where y is the ratio of surviving individuals and T is the temperature of exposure, for fitting the data and calculated the constant (a) and temperature coefficient (b) with a standard error (SE) for each tested species. The function dose.p with the argument p = 0.5 from the library (MASS) in R was used to compute the 50% lethal doses (LD_50_) for the tested species. This function computed the SE for each of the estimated LD_50_ values. The graphs of the relative frequency of survival versus temperature, where the relative portion of surviving individuals was fitted by a logistic curve, were drawn in STATISTICA v. 6.0 (Hill and Lewicki 2006). The differences in LD_50_ between the urban and natural populations, in mean soil temperature and also in elevation between the urban and natural sites were tested using a nonparametric test (Mann-Whitney U test, STATISTICA v. 6.0). The following dependencies of mean soil temperature on elevation, LD_50_ on mean temperature and LD_50_ on elevation were tested using regression analysis (general linear models, STATISTICA v. 6.0).

The mean body length of individuals from urban sites and natural populations was compared using the t-test with separate variance estimates (Basic statistics, Statistica v. 6.0). The significance of the differences in the mean body length between the studied populations was assessed using a nonparametric test (Kruskal-Wallis ANOVA with multiple comparisons of mean ranks, Nonparametrics, Statistica v. 6.0). The relationship between the mean body length and mean soil temperature (annual and the 6-month temperature) was analysed using GLM – Simple regression.

## Results

### Environmental characteristics of the sites

**Figure 2.**
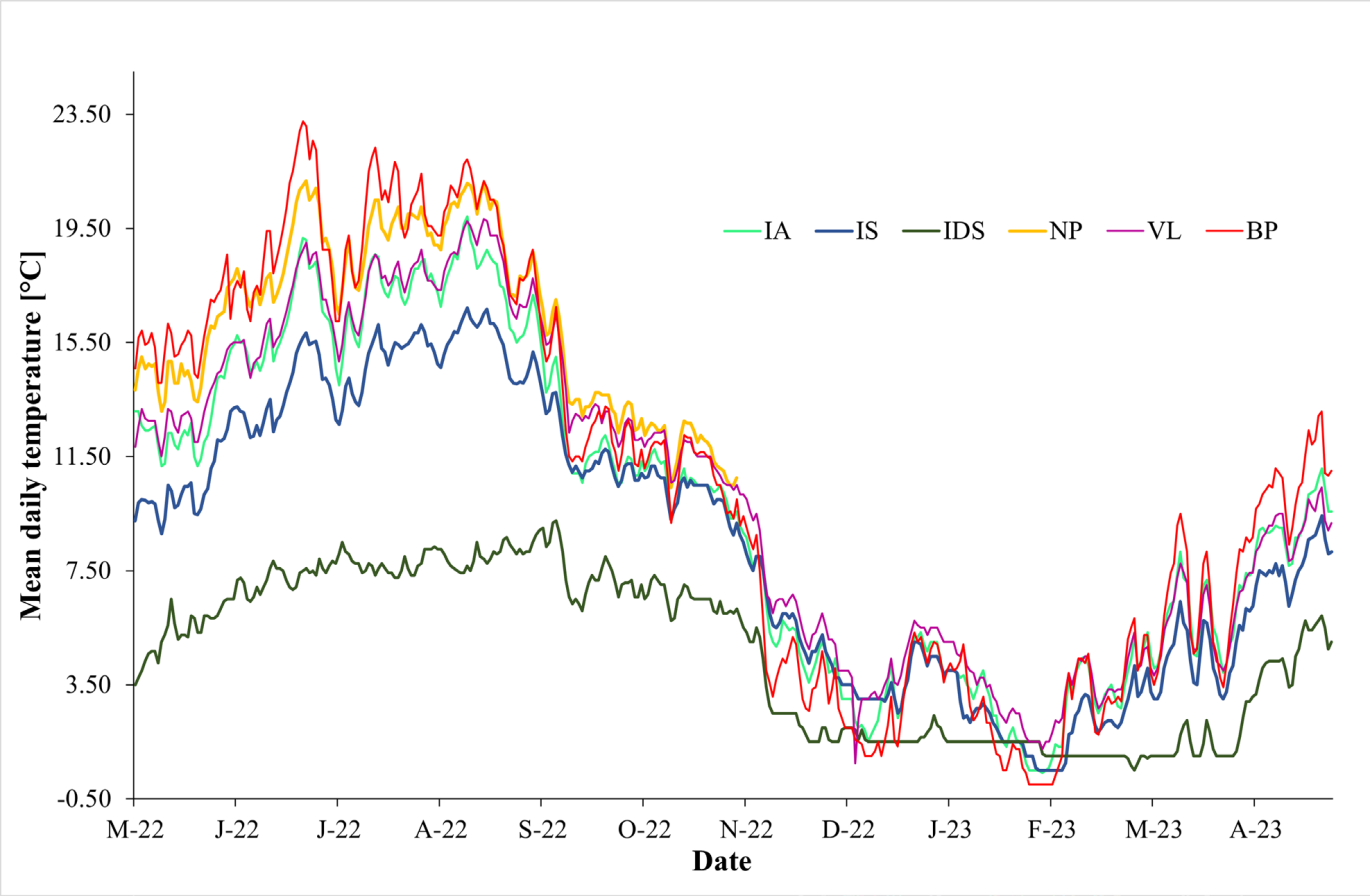
Annual trends of soil temperatures (based on daily averages) at urban and natural sites (for site NP only a 6-month period was available). Site abbreviations: BP – Park (Košice – Barca), NP – Park (Košice), VL – Fragment of woodland (Košice), IA – thermophilous cornel-oak wood, IS – thermophilous hornbeam wood, IDS – coniferous wood.

The topographic and vegetation characteristics as well as the content of soil organic carbon documented differences between the individual sites (Table 1). The maximum difference in elevation between an urban and natural site (BP – IDS) was ~ 773 m a.s.l. In contrast to the urban sites, a dense herbal cover, a thick layer of litter cover and high soil organic carbon content were observed at the natural sites. The soil temperature characteristics at the sites are documented (Table 2, Fig. 2, Suppl. material 1). All soil samples were collected in late autumn before the first snowfall. The mean soil temperatures fluctuated more at the urban sites than at the natural sites. The maximum difference in soil temperature between an urban and natural site (BP – IDS) was ~ 6.1 °C.

Mean temperatures at the urban sites were significantly higher than those at the natural sites (Mann-Whitney U test, Z = –1.963, p = 0.0495). The natural sites were located at higher elevations than the urban sites (Mann-Whitney U test, Z = 1.964, p = 0.0495), and the dependence of mean temperatures on elevation was significant (F_1,4_ = 298.9, p < 0.001). Thus, these results showed that elevation was a strong driver of temperature differences; therefore, it is impossible to evaluate the urbanisation effect separately in our study system.

### Cold tolerance of populations

**Figure 3.**
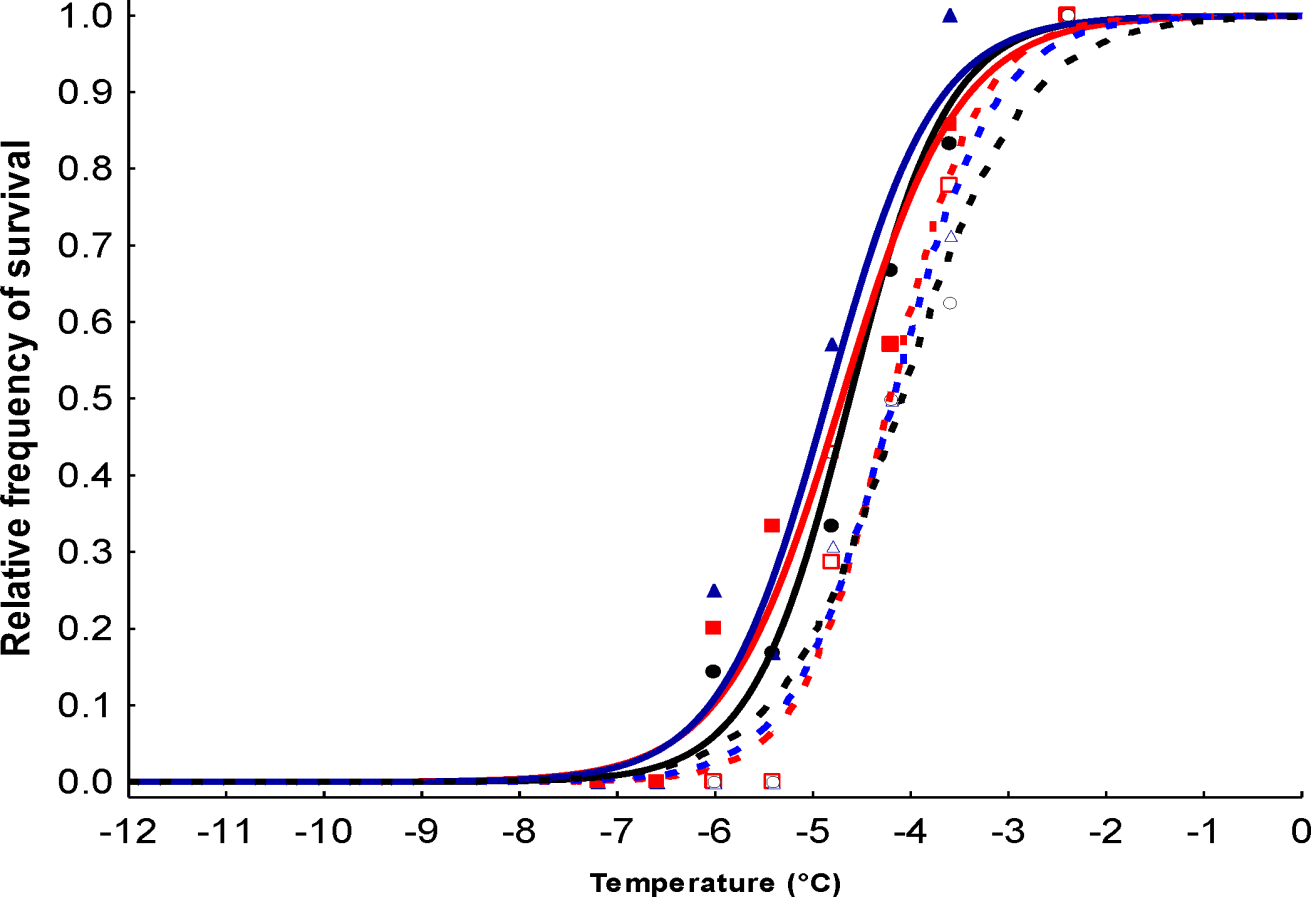
Dependence of the survival rate of natural and urban populations of *Isotomiella minor* on temperature exposure fitted by a logistic curve. Natural populations – full symbols and continuous lines: blue triangles and line = IDS; red squares and line = IA; black circles and line = IS. Urban populations – empty symbols, dashed lines: blue triangles and line = BP; red squares and line = VL; black circles and line = NP. For abbreviations of the sampling sites, see Table 1.

The survival of *I. minor* individuals, tested in the laboratory at different temperatures below zero, is illustrated by the experimental data provided in Fig. 3. Regarding the number of specimens per population, previous studies (e.g., Raschmanová et al. 2015, 2018) have shown that survival curves are reliable with a comparable number of replicates. All populations showed a similar slope of the survival-temperature curve, but in the urban populations the curves were shifted to higher temperatures compared to the natural populations.

**Figure 4.**
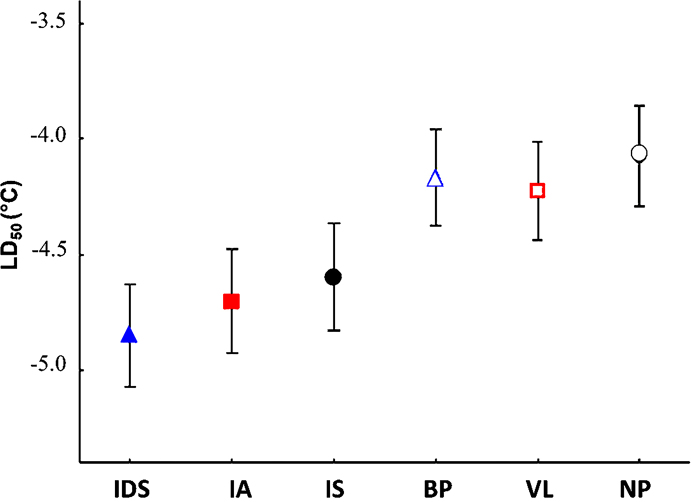
LD_50_ values of natural and urban populations of *Isotomiella minor*. Symbols as in Fig. 3, full symbols = natural populations, empty symbols = urban populations. For abbreviations of the sampling sites, see Table 1; error bars = standard errors (SE).

The LD_50_ values of the natural populations were significantly lower compared to those of the urban populations (Mann-Whitney U test, Z = 1.963, p = 0.0495, Fig. 4). *Isotomiella minor* inhabiting a cold coniferous wood (IDS) was the most cold-resistant population, showing an LD_50_ of –4.9 °C ± 0.2. Populations inhabiting the two natural thermophilous sites, IS and IA, were slightly less cold tolerant than the previous one, with an LD_50_ of –4.7 °C ± 0.2 and –4.6 °C ± 0.2, respectively. Urban populations VL, BP and NP were cold sensitive, showing an LD_50_ of –4.22 °C ± 0.2, –4.17 °C ± 0.2 and –4.07 °C ± 0.2, respectively.

The LD_50_ values were significantly dependent on soil temperature at the sites (F_1,4_ = 9.49, p = 0.037). Regarding site elevation, the dependence of the LD_50_ values remained just below the significance threshold (F_1,4_ = 7.49, p = 0.052).

### Body size of populations

The mean body length of individuals from natural populations was higher than the body length of individuals from urban sites, but the variances of both means were not homogeneous (t-test, p < 0.001, Levene’s test p < 0.005).

**Figure 5.**
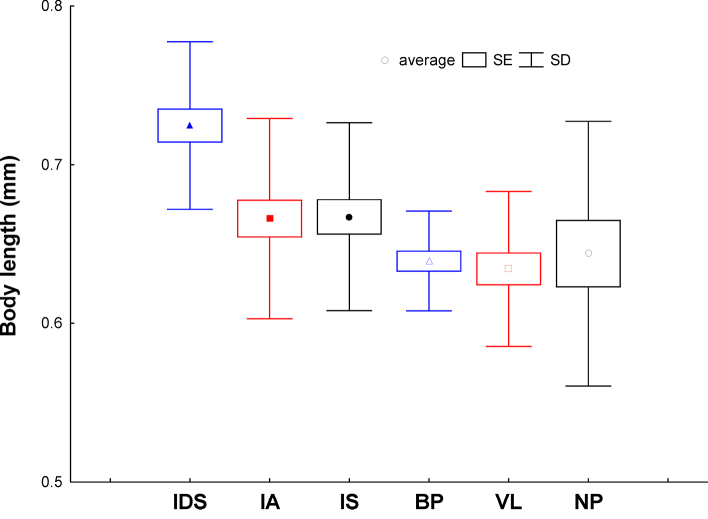
Body length of natural and urban populations of *Isotomiella minor*. Central symbol – mean (symbols as in Fig. 3, full symbols = natural populations, empty symbols = urban populations), the box – SE (standard error) and whiskers – SD (standard deviation). For abbreviations of the sampling sites, see Table 1.

Regarding a comparison of population means, body length was significantly higher in the natural IDS population compared to other populations (Kruskal-Wallis ANOVA with multiple comparisons of mean ranks, p < 0.02). *Isotomiella minor* inhabiting a cold site (IDS) was the largest population, with a mean body length of 0.72 mm ± 0.05 (Fig. 5). Populations inhabiting the two natural thermophilous sites, IS and IA, showed lower values than the previous ones, with a mean body length of 0.67 mm ± 0.06 in both cases. Urban populations NP, BP and VL showed slightly lower values than previous natural populations IS and IA, having a mean body length of 0.64 mm ± 0.08, 0.64 mm ± 0.03, and 0.63 mm ± 0.05, respectively.

**Figure 6.**
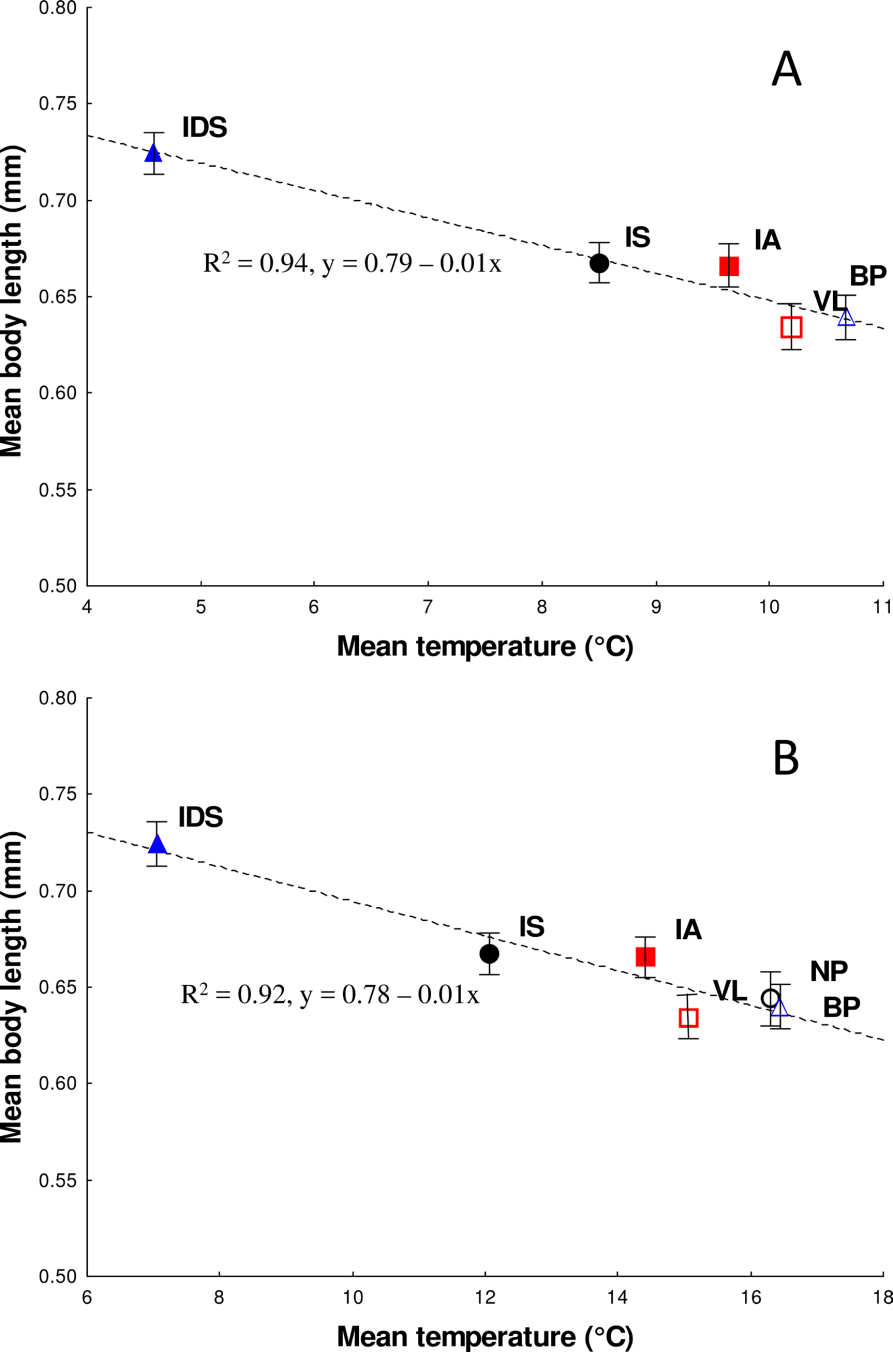
Dependence of the mean body length on habitat (mean) temperature in natural and urban populations of *Isotomiella minor*. **A**. Annual temperature data; **B**. 6-month temperature data. Symbols as in Fig. 3, full symbols = natural populations, empty symbols = urban populations. For abbreviations of the sampling sites, see Table 1. Error bars = standard errors (SE).

In general, mean body length decreased significantly with increasing annual temperature (GLM – Simple regression, F _1,3_ = 50.6, p = 0.006) and 6-month temperature (GLM – Simple regression, F _1,4_ = 43.3, p = 0.003) (Fig. 6A, B).

## Discussion

### Lethal temperatures of *Isotomiella minor* and environmental factors

We found significant differences in the cold survival among *Isotomiella* populations acclimatised to the temperature conditions of their individual habitats. Higher lethal dose values (LD_50_) were observed in populations associated with thermophilous urban sites than in natural forest sites, which is in agreement with our hypothesis. The thermal tolerance of populations significantly correlated with the habitat temperature and were considerably different between the urban and natural sites. Our results indicate that the differences in LD_50_ values between the urban and natural populations may be attributed to the differences in soil temperatures as a consequence of site elevation. A significant relationship between thermal tolerance traits and soil temperature or elevation across populations was also found along environmental gradients (e.g., ants – Tonione et al. 2020). Moreover, this hypothesis was only partially supported by Angilletta et al. (2007) in ants. Considerably high heat tolerance in urban populations was reported in which it took 20% more time to lose mobility at high temperature compared to their rural counterparts. However, this greater heat tolerance in urban populations was not recorded at the expense of cold tolerance. In contrast to previous studies, our urban and natural sites were markedly asymmetrical in terms of elevation, and so the effect of urbanisation on the thermal tolerances of the studied populations could not be fully specified. As noted in previous studies (Imhoff et al. (2010); Stewart and Oke (2012); Diamond et al. (2015)), urban areas exhibit narrower environmental temperature profiles, with increases in mean temperature compared to nearby rural areas. However, the studied urban sites did not show less fluctuating temperatures (narrower temperature profiles) than the natural sites. Only mountain forest at higher elevation was associated with a less fluctuating microclimate. Urban sites had markedly sparse vegetation cover in summer and a thin snow cover in winter compared to the natural sites. Although habitat temperature in combination with topography and vegetation seems to exert a significant evolutionary pressure on *Isotomiella* populations in this habitat complex, other environmental factors which were not included/considered in this study, such as soil moisture, soil type or gut content, could also have an influence on the cold tolerance of the studied populations (Sømme 1982; Hass et al. 2016; Wehrli et al. 2025).

Generally, Collembola are a freeze-intolerant invertebrates, with a strategy of the chemical lowering of the supercooling point of the body fluid (Sømme 1982; Sinclair et al. 2003). The low lethal temperature may be considered to be higher or equal to their SCP (Sinclair et al. 2015). Although it is unclear what specific strategies *I. minor* uses under low temperatures to avoid freezing, it is evident that this soil dwelling (euedaphic) isotomid, occupying less temperature fluctuating environments compared to epedaphic species, can easily migrate vertically into lower soil horizons during low temperature conditions or may use overwintering strategies in the form of eggs or survival in quiescence (Zettel 1999/2000; Salmon et al. 2014). Zettel (1984) showed that a population of *I. minor* inhabiting subalpine forests had a significantly higher degree of thermal hysteresis in winter compared to summer period. This means that this species is probably protected against freezing during a longer period. The study also suggested that seasonal acclimatisation is possible in this species. In our study, the lethal dose in all populations of *I. minor* was generally lower than the annual temperature minimum at the sites, indicating that they have sufficient physiological capacity to survive temperature minima. However, we are aware that laboratory acclimatisation is also needed to conduct reliable observations (Hawes et al. 2006).

The differences in cold tolerance found in *I. minor* imply that its populations from contrasting environments may have different responses to global warming. In this context, populations inhabiting a cold and relatively high-elevation site in a less climatically fluctuating environment will be more buffered from thermal changes than others living in urban parks with a thermally fluctuating microclimate. Generally, higher variations in maximum temperatures and the frequency of temperature extremes are important in the context of species extinction due to climate change (Roman-Palacios and Wiens 2020).

Sinclair et al. (2015) pointed out that the data on cold hardiness from different studies are usually incomparable due to diverse experimental designs in studies (cooling rates, exposure, and recovery times). In this regard, the present study has two advantages. Firstly, the experimental design can be applied to compare observed data with previous studies (Raschmanová et al. 2015, 2017, 2018) since a uniform methodology for cold tolerance tests was used. Secondly, these studies also included non-laboratory springtail species. In this context, isotomid *I. minor* tested in the present study showed the weakest tolerance to low temperatures than 18 experimentally tested Collembola species in the previous studies (Raschmanová et al. 2017: fig. 3; Raschmanová et al. 2018: Table 2). The weak motion of the specimens was observed at higher temperatures during the set of cold measurements, indicating its susceptibility to relatively low values. However, *I. minor* as a species with wide ecological valence and broad geographical distribution, should tolerate greater thermal ranges. This species can be more tolerant to high rather than low temperatures, showing higher heat tolerance and thus a broader temperature tolerance range.

### Body size and temperature

The mean body length of individuals from warmer urban sites was lower than at colder natural sites. More precisely, we found that *Isotomiella minor* inhabiting the mountain forest site at a higher elevation was significantly larger than other populations, i.e., from the thermophilous forest sites and urban sites of lower elevation. The body size of *Isotomiella* populations decreased significantly with increasing habitat temperature and decreasing elevation. Also, Sun et al. (2020b) showed that body size in representatives of the subfamily Onychiurinae species increases with increasing elevation. A variety of possible explanations have been proposed for the relationship between temperature and body size in ectothermic organisms. There is no general and simple explanation for the Atkinson’s temperature-size rule (Angilletta and Dunham 2003), which states that a temperature increase in the environment is associated with decreasing body size at the organism level. In addition to temperature, other abiotic factors, such as organic carbon, vegetation structure, moisture content, but also developmental time, metabolic rate and resource availability, can influence invertebrate body size (e.g.. Gray 1989; Atkinson 1994; Magura et al. 2004; Chown and Gaston 2010; Lowe et al. 2014; Andriuzzi and Wall 2018). For instance, studies focused on terrestrial arthropods inhabiting urban and rural areas along marked disturbance gradients have shown that the body size of soil beetles in rural areas was significantly larger than in urban environments (e.g., Gray 1989; Magura et al. 2004). Increased disturbance associated with the lower quality and availability of food at urban sites had a significant impact on this pattern. In contrast to previous studies, Sadler et al. (2006) found different pattern, carabid species were larger in urban habitats than in nearby rural sites, which was related to a greater availability and/or quality of food, or a lower interspecific competition.

In the present study, a markedly denser vegetation cover, a thicker litter layer and a greater amount of decaying wood material were observed at the natural sites than at the urban sites (tree parks, woodland fragment). As the result, a significantly higher content of soil organic carbon was found at the natural forest sites than at the urban sites. On the other hand, urbanisation, in combination with temperature and vegetation, may also have a different effect on invertebrate body size. Warmer temperatures, a longer growing season and greater resource availability at urban sites may lead to opposite ecological patterns in the relationship between temperature and body size (Lowe et al. 2014). Based on this observation, we could expect a smaller body size in individuals of a given population at natural sites than at urban sites. Regardless, our study did not specifically focus on the effect of urbanisation on the body size of *Isotomiella* populations; that remains to be considered in the future studies. Finally, it may be generalised that temperature of the environment, in combination with other factors, is among the critical factors affecting body size in Collembola. However, studies based on larger datasets from northern and southern European regions, from various urban and natural habitats and including different species and life forms of Collembola (e.g., Sun et al. 2020a, 2020b; Xie et al. 2024), are required to verify and clarify this complex relationship. Moreover, genetic studies may help to shed more light on the body size variation of parthenogenetic species. Parthenogenetic reproduction may influence body size patterns through clonal lineages. For instance, different body size of clonal lineages from contrasting environments, such as forests and grasslands, was observed in a parthenogenetic and ubiquitous soil oribatid mite (von Saltzwedel et al. 2014). Additionally, genetic lineages specifically adapted to these contrasting environments were identified. In our previous study (Fedičová et al. 2025) several cryptic lineages in parthenogenetic *Isotomiella minor* were observed in this habitat complex, and these could differ in body size. However, the connection between genetic lineages and morphological differentiation of urban and natural *Isotomiella* populations requires further investigation.

In the final, we cannot infer whether the difference between the cold tolerances of urban and natural *Isotomiella* populations are determined genetically in terms of evolutionary adaptation or simply result from a physiological adaptation (acclimatisation) as a manifestation of phenotypic plasticity, i.e. the ability of a genotype to give rise to different phenotypic expressions under variable environmental conditions (e.g., Smith and Smith 2015). Testing genetically fixed evolutionary adaptations requires further molecular approaches, such as cold hardiness influencing functional genes presence and transcription or proteomics. Testing the physiological adaptability of different populations requires experiments with acclimation under controlled conditions based on successfully reproducing laboratory cultures. For small, euedaphic collembolan species, such phenotypic plasticity studies are scarce due to difficulties in sustaining laboratory cultures (Raschmanová et al. 2017). Regarding body size differences, there is evidence that primarily phenotypic plasticity is observed across urbanisation gradients in arthropods (Austin et al. 2022). Alternatively, some studies have outlined genetic adaptations to urban environments (Diamond et al. 2022), showing that both cases lead to intraspecific trait variation in body size in urban-natural systems.

## Conclusions

Cold tolerance and body size were examined in the common springtail *Isotomiella minor* from urban and natural environments differing in microclimate and topography. Our study outlined that populations of parthenogenetic, generalist springtail species with wide geographic distributions and inhabiting different environments with varying environmental conditions, may show remarkable differences in physiological and morphological traits. Habitat temperature in combination with other environmental characteristics seems to exert a significant evolutionary pressure on *Isotomiella* populations in contrasting landscapes, in terms of these traits. Revealing the thermal tolerance and body size in populations of *Isotomiella minor* may suggest their ecological resilience to global climate changes in contrasting environments. It is expected that urban agglomerations will experience progressing temperature increase, and the narrow thermal tolerances of species inhabiting these urban areas could narrow even further under global warming. Most probably, such responses can be expected, but probably after a longer period since soil fauna generally show high resilience to soil warming (>20 years, e.g., Alatalo et al. 2015). Furthermore, our study is limited by including relatively low number of populations of *I. minor*. It thus represents an initial step towards investigating cold tolerance and body size in this ubiquitous species in habitats with contrasting environmental conditions.

## Additional information

### Use of AI

No use of AI was reported.

### Author ORCIDs

Natália Raschmanová https://orcid.org/0000-0002-9476-1753

Vladimír Šustr https://orcid.org/0000-0003-2983-1855

Mária Fedičová https://orcid.org/0009-0000-2109-7567

Michal Hurka https://orcid.org/0009-0009-4652-572X

Martina Žurovcová https://orcid.org/0000-0003-1539-9774

Ľubomír Kováč https://orcid.org/0000-0001-8194-2128

### Data availability

All of the data that support the findings of this study are available in the main text or Supplementary Information.

## Supplementary materials

Supplementary material 1 Temperature shapes the cold tolerance and body size of the springtail *Isotomiella minor* (Hexapoda, Collembola) in contrasting environments Data type docx Explanation note Temperature characteristics of the sites - mean, minimum and maximum temperatures. https://binary.pensoft.net/file/1589175This dataset is made available under the Open Database License (http://opendatacommons.org/licenses/odbl/1.0/). The Open Database License (ODbL) is a license agreement intended to allow users to freely share, modify, and use this Dataset while maintaining this same freedom for others, provided that the original source and author(s) are credited. Natália Raschmanová, Vladimír Šustr, Mária Fedičová, Michal Hurka, Martina Žurovcová, Ľubomír Kováč
