# Supplementary material for: Temperature shapes the cold tolerance and body size of the springtail Isotomiella minor (Hexapoda, Collembola) in contrasting environments
Source: Zookeys. 2026 Apr 9;1277:57–78. doi: 10.3897/zookeys.1277.172390 (PMC13087656; doi:10.3897/zookeys.1277.172390)
Supplement: Supplementary file 1 [file zookeys-1277-057_article-172390__-s001.docx]

Supplementary material 1

Title: Temperature shapes the cold tolerance and body size of the springtail Isotomiella minor (Hexapoda, Collembola) in contrasting environments

Authors:          Natália Raschmanová1*, Vladimír Šustr2, Mária Fedičová1, Michal Hurka1, Martina Žurovcová3, Ľubomír Kováč1 

1Department of Zoology, Institute of Biology and Ecology, Faculty of Science, Pavol Jozef Šafárik University, SK-04154 Košice, Slovakia,

2Institute of Soil Biology and Biogeochemistry, Biology Centre AS CR v. v. i., Na Sádkách 702/7, CZ-37005 České Budějovice, Czech Republic,

3Institute of Entomology, Biology Centre AS CR v. v. i., Branišovská 31, CZ-37005 České Budějovice, Czech Republic

- Corresponding author: [natalia.raschmanova@upjs.sk](mailto:natalia.raschmanova@upjs.sk) (N. Raschmanová)

Temperature characteristics of the sites

Differences in soil temperature between the urban sites were smaller than those between the natural sites, 0.5 °C and 1.1–5.0 °C, respectively; during the 6-month vegetation period: 0.2–1.4 °C and 2.3–7.3 °C, respectively. At all sites, a temperature minimum of 0/0.5 °C was recorded (February). In contrast to the natural sites, a thin snow cover was observed at the urban sites. Therefore, soil temperatures at the natural thermophilous sites, IA and IS, were similar to those at the urban sites VL and BP in winter. The highest temperature maximum of 22.5/25 °C was reported in the city parks (June).
